# Modelling the dynamics of two political parties in the presence of switching

**DOI:** 10.1186/s40064-016-2483-z

**Published:** 2016-07-08

**Authors:** F. Nyabadza, Tobge Yawo Alassey, Gift Muchatibaya

**Affiliations:** Department of Mathematical Science, University of Stellenbosch, Private Bag X1, Matieland, 7602 South Africa; African Institute of Mathematical Sciences, Birwa, Ghana; Department of Mathematics, University of Zimbabwe, P. Box MP 167, Mount Pleasant, Harare, Zimbabwe

**Keywords:** Political parties, Modelling, Switching function, Steady states, Simulations

## Abstract

This paper generalizes the model proposed by Misra, by considering switching between political parties. In the model proposed, the movements of members from political party *B* to political party *C* and vice versa, are considered but the net movement is considered by assuming that $\theta _1-\theta _2=\theta$ (a constant), which implies that the movement of members is either from party *B* to party *C* or from party *C* to party *B*. In this paper we remodel these movements through switching functions to capture how individuals switch between parties. The results provide a more comprehensive synopsis of the dynamics between two political parties.

## Background

In ecology, the term switching was first coined by Murdoch in 1969, to describe a scenario where a predator predominantly eats the most common type of prey, see Murdoch (1969) and is often accompanied by a change in the habitat Khan (2000). Prey switching however happens when a predator’s preference for a particular type of prey increases as the prey increases in abundance. Any display by a predator, of prey switching behaviour, can significantly affect the stability of the system, coexistence of prey species and evolutionary diversification. Switching can however promote coexistence between prey species Abrams and Matsuda (2003). A classical example is the case where prey switching causes low predation for rare prey, thus aiding prey refugia that often leads to coexistence Gentleman et al. (1990).

More often than not, political parties compete for membership. Members often switch between political parties as preferences change, often as a result of change of leadership, policies and perceived gains Fieldhouse et al. (2007), Petersen (1991), Schofield and Sened (2005), Romero et al. (2009). This paper is motivated by the work in Misra (2012). A closer loot at the work in Misra (2012) shows that there were simplifying assumptions that made the mathematical model tractable but overlooking some essential elements such as switching. The parameters $\theta _1$ and $\theta _2$ model movements between political parties *B* and *C*. The net shifting of members $\theta =\theta _1-\theta _2$ is considered to be constant resulting in a unidirectional movement of members from *B* to *C* and vice versa. In this paper, we relook at this assumption by introducing switching functions whose parameters are endogenous to the system.

The paper is arranged as follows: in “The Misra model” section, we generalize the Misra model by including switching functions. The stabilities of the steady states are presented in “Stability of steady states” section and the paper is concluded in “Conclusion” section.

## The Misra model

The model uses principles of mathematical epidemiology to model the dynamics of the two political parties. In general, most dynamical social phenomenon may be modelled by using these epidemiological type differential equations, see for instance Petersen (1991), Alvarez and Nagler (2000), Burden (2004), Huckfeldt and Kohfeld (1992), Belenky and King (2007). Following Misra (2012), the model is based on the following system of equations:

1 $$\begin{aligned} \dfrac{dV}{dt} & = \mu N - \beta _1 V\dfrac{B}{N} - \beta _2 V\dfrac{C}{N}- \mu V, \\ \dfrac{dB}{dt} & = \beta _1 V\dfrac{B}{N} - \theta _1 B\dfrac{C}{N} + \theta _2 C\dfrac{B}{N}-\mu B, \\ \dfrac{dC}{dt} & = \beta _2 V\dfrac{C}{N} + \theta _1 B\dfrac{C}{N} - \theta _2 C\dfrac{B}{N}-\mu C. \end{aligned}$$

Here, the total number of population *N*(*t*) which was assumed constant, was divided into three classes, namely; voters class *V*, political party *B* and political party *C*. The parameters and model assumptions are given in Misra (2012).

The non-dimensionalised model was obtained by setting

$$\begin{aligned} v = \frac{V}{N} , \quad b = \frac{B}{N} ,\quad \text {and} \quad c = \frac{C}{N}, \end{aligned}$$

so that

2 $$\begin{aligned} \dfrac{dv}{dt}& = \mu - \beta _1 vb - \beta _2 vc - \mu v, \\ \dfrac{db}{dt} & = \beta _1 vb - (\theta _1 - \theta _2)bc - \mu b, \\ \dfrac{dc}{dt}& = \beta _2 vc + (\theta _1 - \theta _2)bc - \mu c. \end{aligned}$$

The model was then reduced to a 2-dimensional system. One of the simplifying assumption made in the paper was setting $\theta =\theta _1-\theta _2>0.$ This then resulted in a system in which the individuals moved from party *B* to party *C*.

In this note we revisit the model in Misra (2012) and consider the following functions

$$\begin{aligned} \theta _1(b) = \dfrac{\hat{\theta }_1(1-e^{-\alpha _1b})}{1+ me^{-\alpha _1b}} \quad \text {and} \quad \theta _2(c) = \dfrac{\hat{\theta }_2(1-e^{-\alpha _2c})}{1+ me^{-\alpha _2c}}, \end{aligned}$$

where  $\alpha _1, \alpha _2, \,m, \,\hat{\theta _1}$ and $\hat{\theta _2}$  are positive constants, to capture the aspects of switching. The parameters $m, \alpha _1$ and $\alpha _2$ must be chosen such that $\theta _1$ and $\theta _2$ approach 1 when *b*, *c* approach 1. The parameter *m* affects the position of the switching point and $\alpha _1, \alpha _2$ affect both the switching point and the rate at which the switching occurs.

**Fig. 1:**
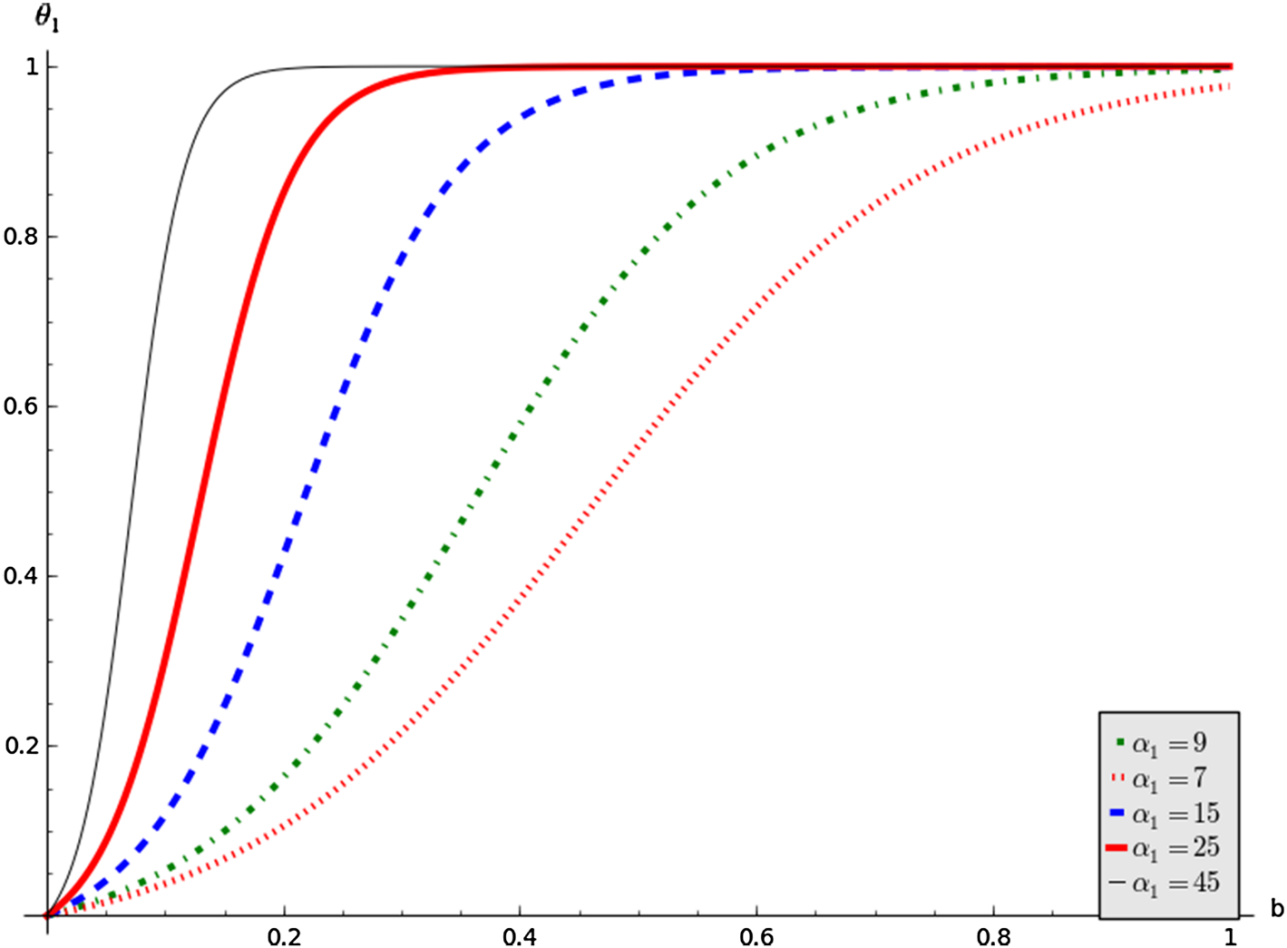
Graphs of  $\theta _1$  against  *b*  for different values of  $\alpha _1$

A graph of the function $\theta _1$ for different values of $\alpha _1$ with $\hat{\theta }_1= 1$ and $m = 25$ is depicted by Fig. 1.

Figure 1 shows switching increases with increasing values of $\alpha _1.$ It is important to note that the graph of $\theta _2$ follows a similar pattern. The gradient function of $\theta _1$ is:

$$\begin{aligned} \dfrac{d\theta _1}{db}=\dfrac{\hat{\theta }_1\alpha _1(1+m)e^{-\alpha _1b}}{(1+ me^{-\alpha _1b})^2}. \end{aligned}$$

**Fig. 2:**
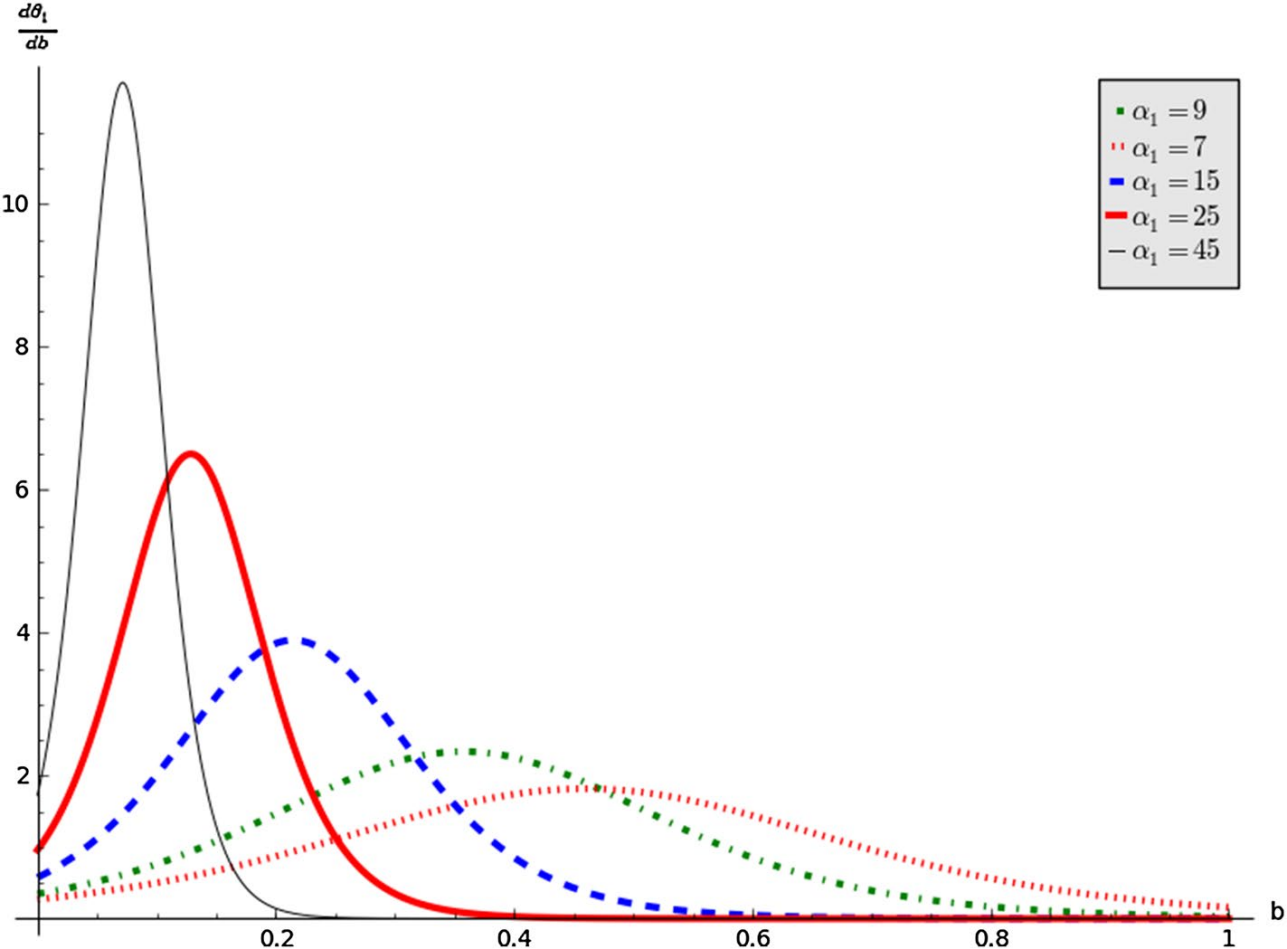
Graphs of $\dfrac{d\theta _1}{db}$ against *b* by varying $\alpha _1$, and setting $\hat{\theta }_1 = 1$ and $m=25$

This function gives the change of $\theta _1$ with respect to *b*. This shows how the switching changes with respect to the state variable. Figure 2 shows the change in switching with respect to *b* for the chosen set of parameter values in the caption. The peak increases with increasing $\alpha _1$. This means that people leave party *B* for party *C* faster and after a while decreases. This is consistent with either Type I or Type II response functions in ecology.

Setting $\theta (t,b,c)=\theta _1 - \theta _2 = \dfrac{\hat{\theta }_1(1-e^{-\alpha _1b})}{1+ me^{-\alpha _1b}} - \dfrac{\hat{\theta }_2(1-e^{-\alpha _2c})}{1+ me^{-\alpha _2c}},$ system (2) reduces to

3 $$\begin{aligned} \dfrac{db}{dt}=\beta _1 (1- b - c)b - \theta (t,b,c) bc - \mu b,\quad \dfrac{dc}{dt} = \beta _2 (1-b - c)c + \theta (t,b,c) bc - \mu c. \end{aligned}$$

Here $\theta (t,b,c)$ can either be positive or negative, thus allowing individuals to switch between political parties. Just as in Misra (2012), system (3) has four equilibria, a party free equilibrium $E_0 = (0,0),$ single party equilibria $E_1 = (1-\dfrac{\mu }{\beta _1},0)$ and $E_2 = (0,1-\dfrac{\mu }{\beta _2}),$ whose existence is subject to $\beta _1 > \mu$ and $\beta _2 > \mu$ respectively and the interior equilibrium. Unlike in Misra (2012), the interior equilibrium is only unique for the case $\theta _1(b) =\theta _2(c)$.

**Fig. 3:**
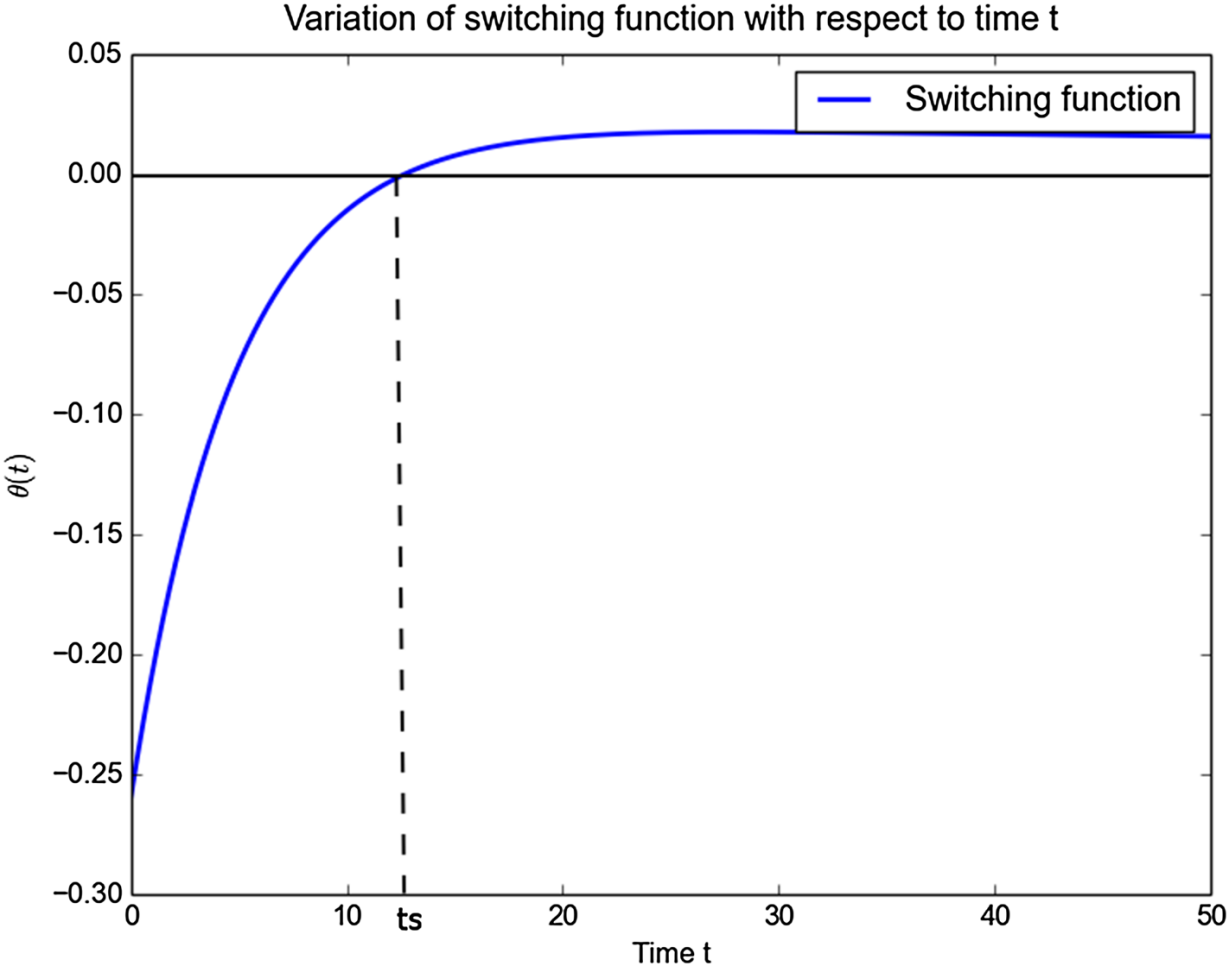
Graphs of the switching function with respect to time

The graph of the switching function $\theta (t,b,c)$ against time is shown in Fig. 3. It is important note that the switching function is an increasing function that is initial negative until time *ts* and then becomes positive there after. The interior equilibrium depends on the sign of switching function $\theta (t)$.

We consider three possible scenarios:

1. The case $\theta (t,b,c)<0.$ This is the case where members leave political party *C* for *B*, for all $t<ts$.
2. The case $$\theta (t,b,c) = 0.$$
3. The case $\theta (t,b,c)>0.$ This is the case considered in Misra (2012). This is the case where members leave political party *B* for *C*, for all $t>ts$ where *ts*  is the time at which the switch occurs.

## Stability of steady states

The stability of the boundary equilibria are presented in Misra (2012). If $\theta (t,b,c) = 0,$ we have

4 $$\left\{ \begin{aligned} \beta _1(1-b^*-c^*) - \mu = 0,&\\ \beta _2 (1-b^*-c^*) - \mu = 0,&\\ \end{aligned} \right. \Longrightarrow b^* + c^* = 1 - \dfrac{\mu }{\beta _1} \quad \text {and} \quad b^* + c^* = 1 - \dfrac{\mu }{\beta _2}.$$

From this system (4), the values of $b^*$ and $c^*$ are obtained as follows:

- If $\beta _1 \ne \beta _2$, then the interior equilibrium $E(b^*,c^*)$  does not exist.
- If $\beta _1 = \beta _2$, then the interior equilibrium is stable and is a straight line satisfying the equation
  $$\begin{aligned} \left\{ b^*, c^* \in [0,1]|b^* + c^* = 1 - \dfrac{\mu }{\beta _1}\right\} . \end{aligned}$$

**Fig. 4:**
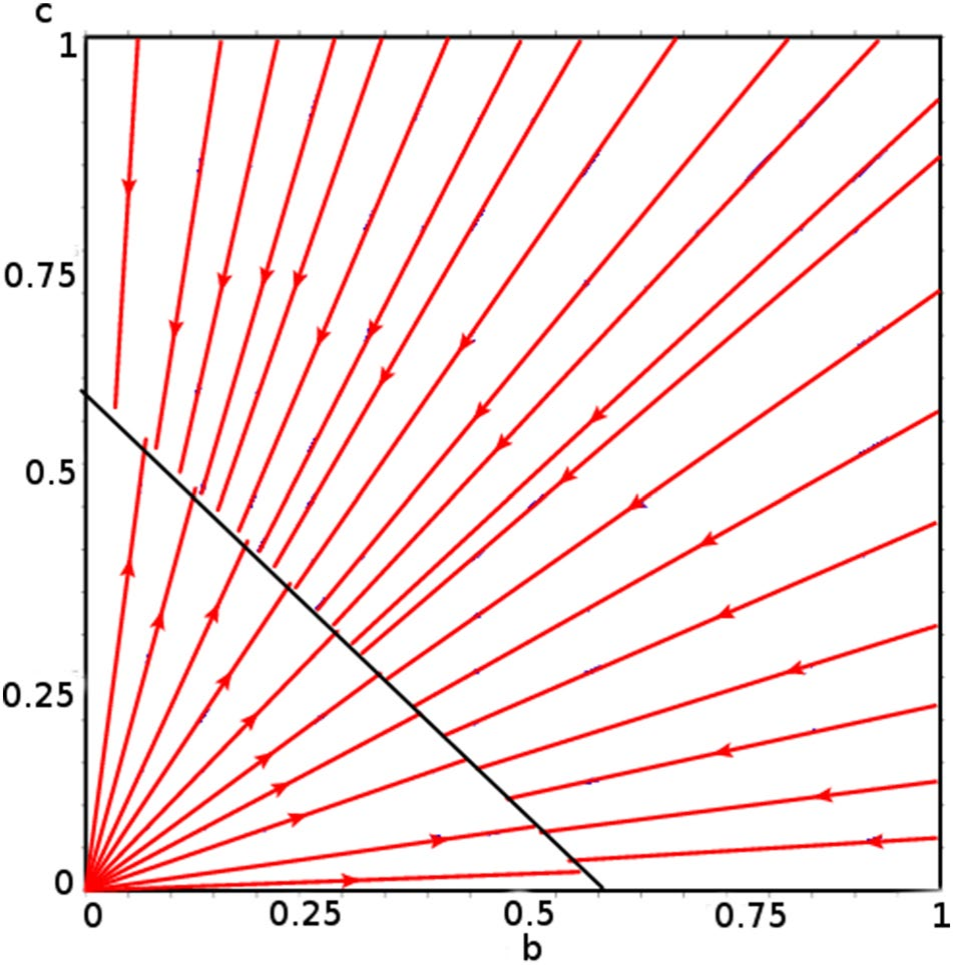
$$\beta _1 = \beta _2 = \dfrac{1}{24}$$

The existence of the interior equilibrium is numerically given in Fig. 4.

**Fig. 5:**
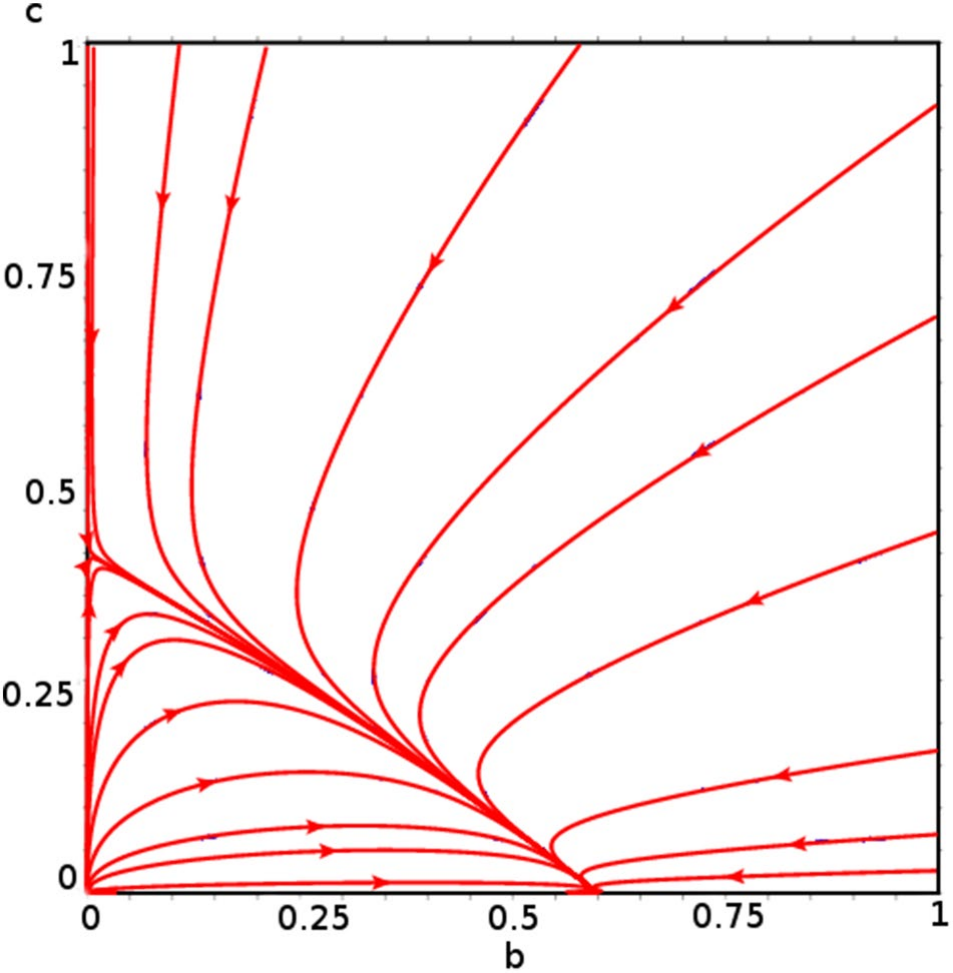
$$\beta _1 \not = \beta _2 , \beta _1= \dfrac{1}{24}, \beta _2=\dfrac{1}{36}$$

Figure 4 shows the existence of the stable interior equilibrium which is a straight line that satisfies the equation

$$\begin{aligned} \left\{ b^*,c^*\in [0,1]|b^* + c^* = 1 - \dfrac{\mu }{\beta _1}\right\} \quad {\hbox{when}} \quad \beta _1 = \beta _2, \end{aligned}$$

whereas Fig. 5 shows the non-existence of the interior equilibrium when $\beta _1 \ne \beta _2.$

We now consider the case when $\theta (t,b,c)\ne 0.$ We begin by considering the plot in which political parties *B* and  *C*  coexist in a stable state. We come up with this result and both political parties start with an initial value of 0.2 at  $t=0$.

**Fig. 6:**
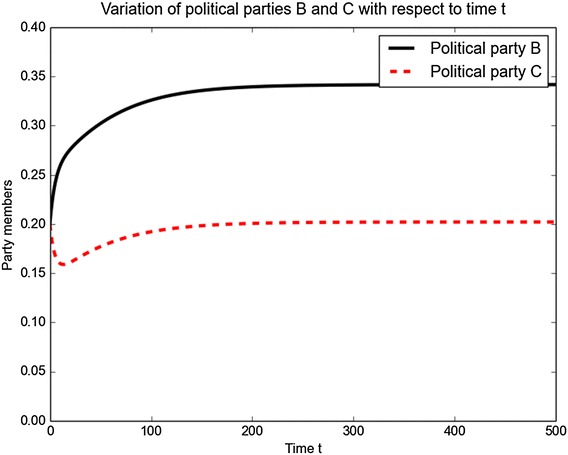
Time series plot for parties  *B*  and  *C*

**Fig. 7:**
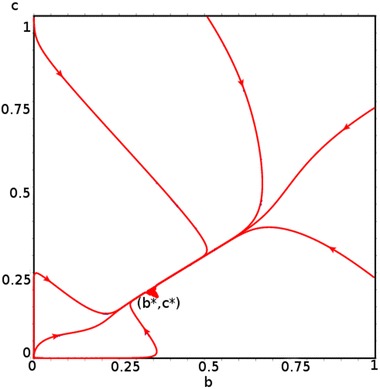
Phase plot for the interior equilibrium $E(b^*,c^*)$

A closer look at the Fig. 6 shows that there exists a time interval $t<ts$ where members of political party *C* leave for political party *B* when $\theta (t)<0$. After that members of political party *B* leave for party *C* before both become stable over the time. The corresponding interior equilibrium is shown in Fig. 7.

**Fig. 8:**
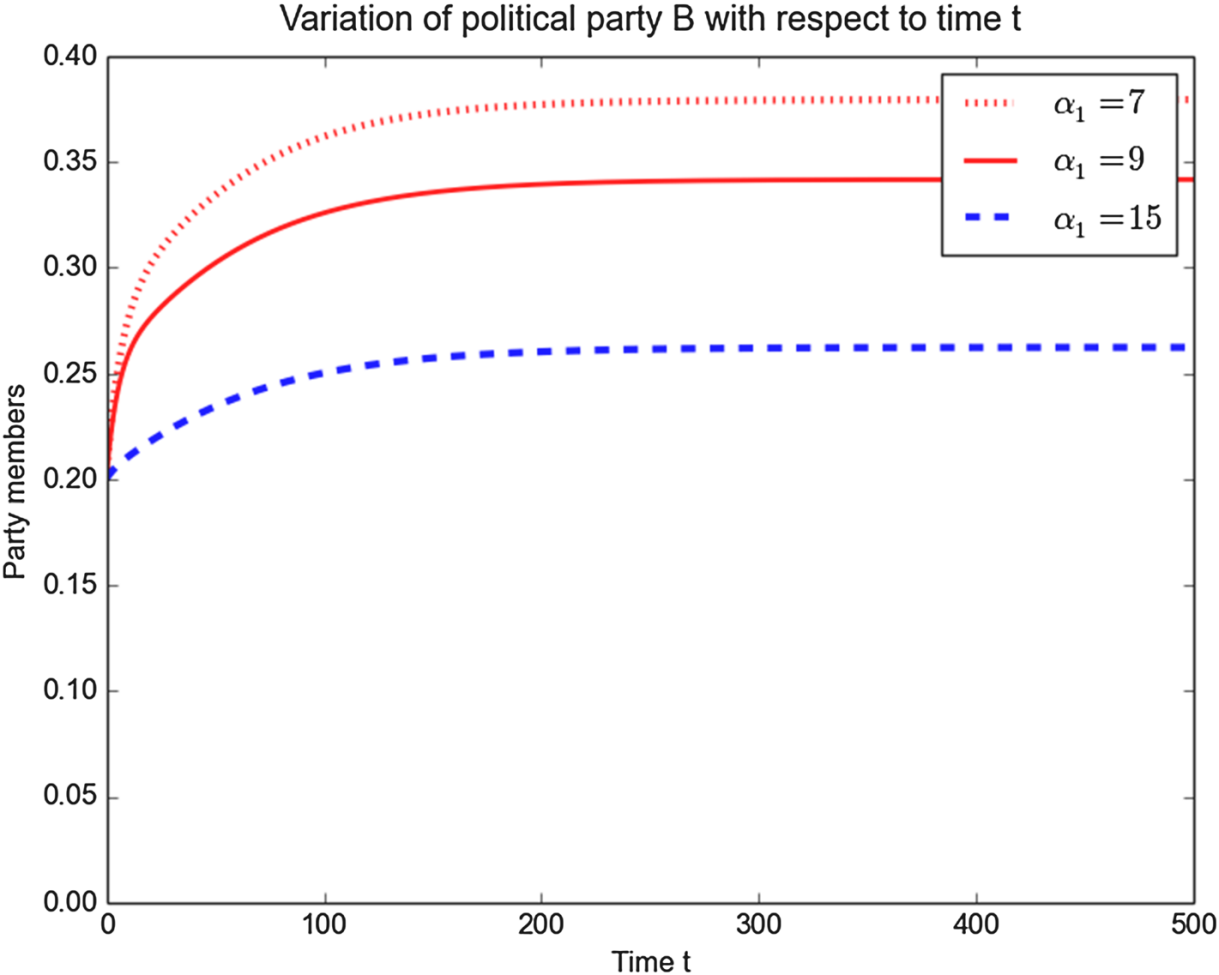
Variation of *B* with *t* for different values of $\alpha _1$

**Fig. 9:**
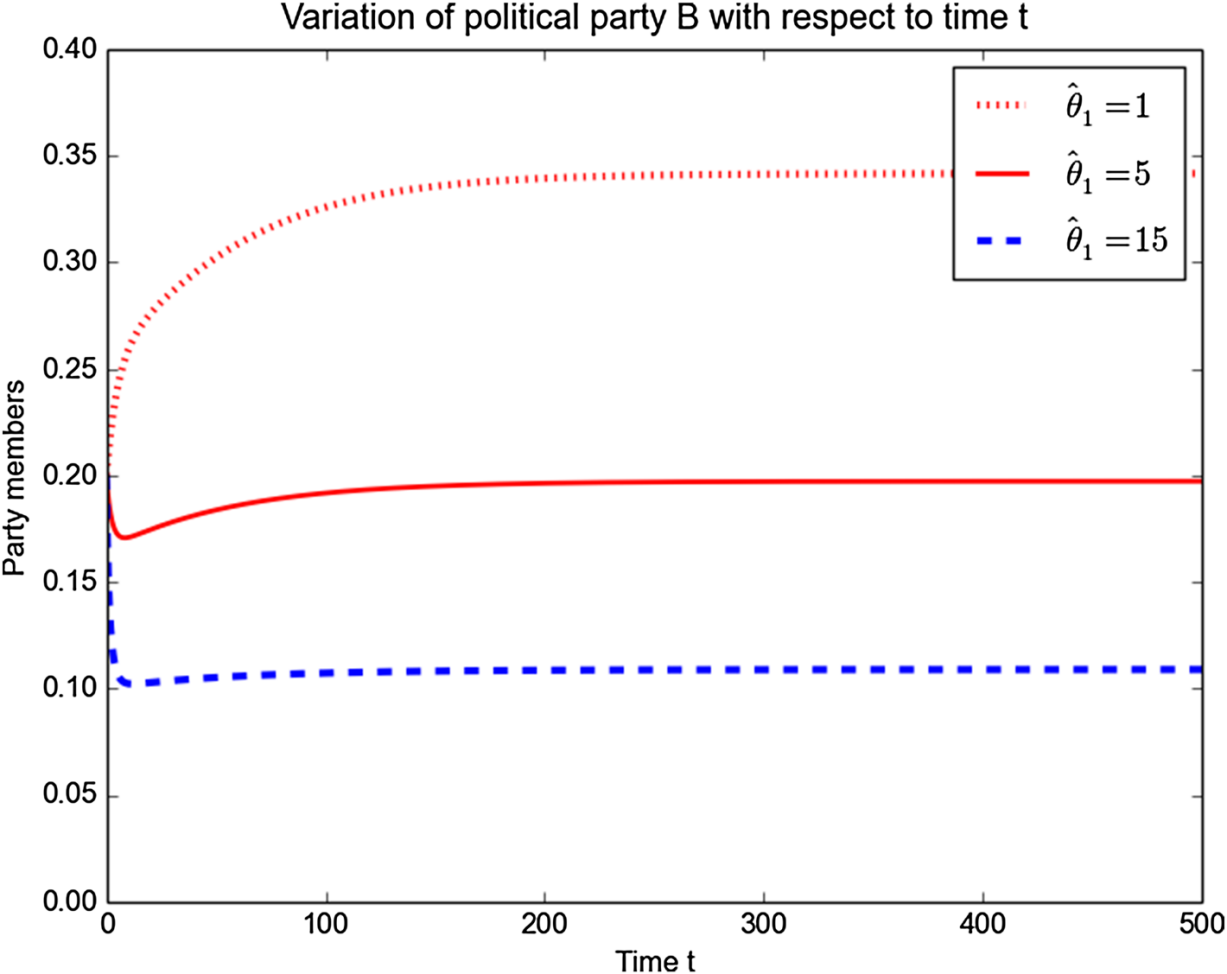
Variation of *B* with *t* for different values of $\hat{\theta }_1$

**Fig. 10:**
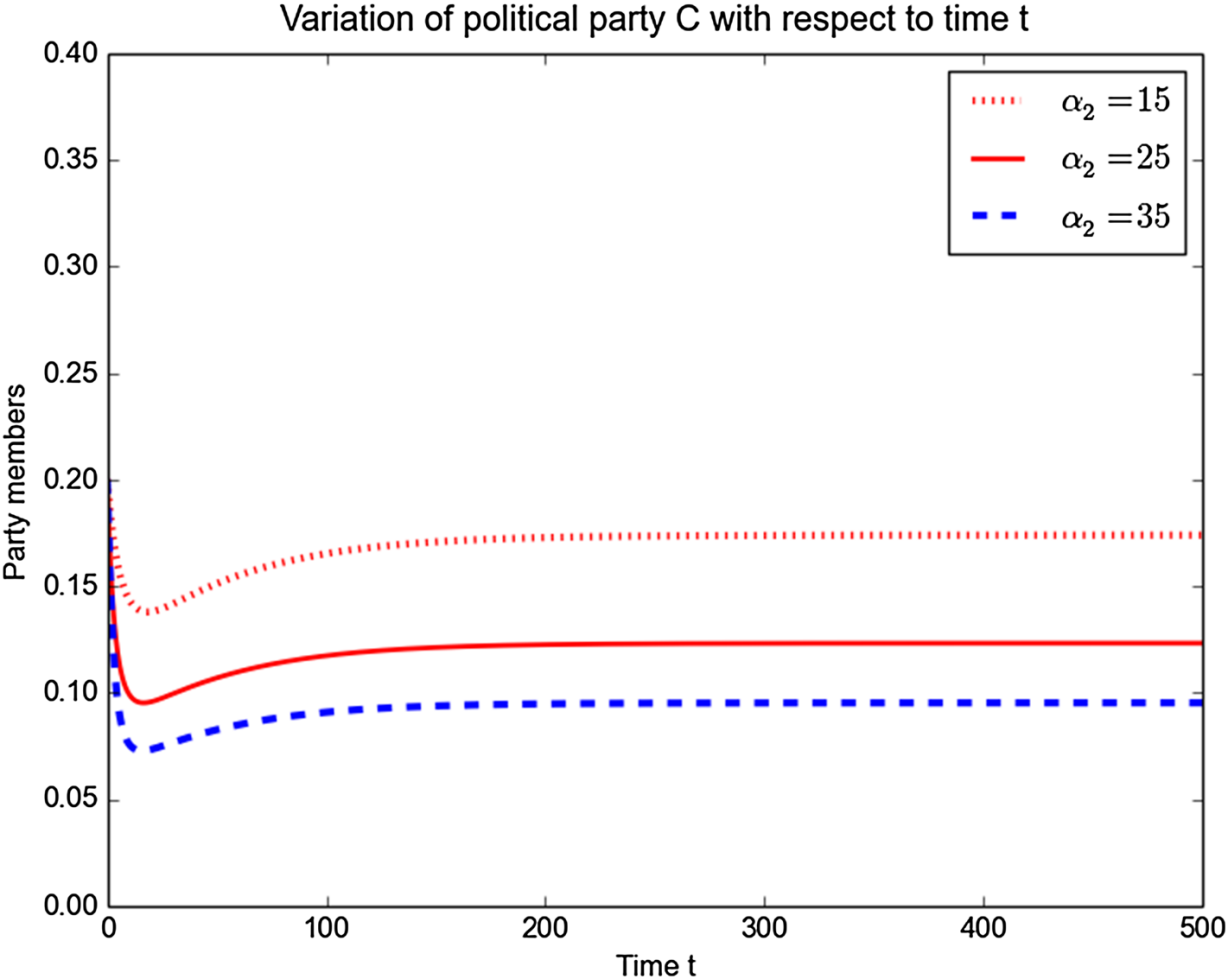
Variation of *C* with *t* for different values of $\alpha _2$

**Fig. 11:**
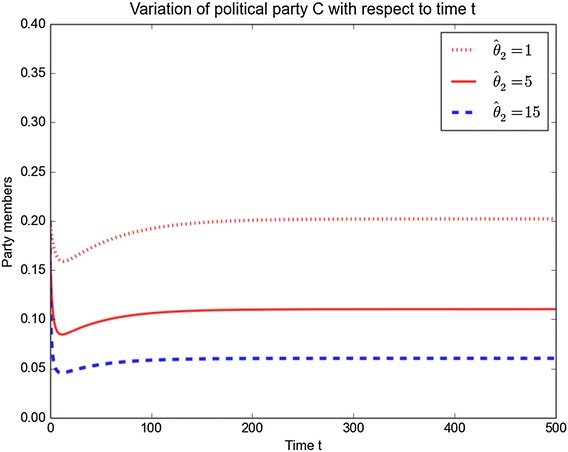
Variation of *C* with *t* for different values of $\hat{\theta }_2$

An interesting aspect to consider is the switching by individuals that take place between political parties. To investigate such dynamics, we plot the variations of parties *B* and *C* as the parameters $\alpha _1, \hat{\theta }_1, \,\alpha _2,$ and $\hat{\theta }_2$ are varied. The results are depicted in Figs. 8, 9, 10 and 11. One can easily observe that increasing $\alpha _1$ and $\alpha _2$ leads to a decrease of membership in both political parties. So to maintain their numbers, political parties should focus on minimising any switching that can take place. A similar result is obtained for $\hat{\theta }_1$ and $\hat{\theta }_2$. Figures 8 and 9 show the evolution of political *B* over the time by varying the parameter values $\alpha _1$ and $\hat{\theta }_1$ while keeping the other parameters constant. The figures show that when increasing the parameter values $\alpha _1$ and $\hat{\theta }_1$, the number of members of political *B* decrease over the time.

Similar plots for political party *C* for different values of $\alpha _2$ and $\hat{\theta }_2$ are shown in Figs. 10 and 11. The figures show that when increasing the parameter values $\alpha _2$ and $\hat{\theta }_2$, leads to a decrease in the number of members of political party *C* over the time.

## Conclusion

In this paper, we remodelled switching between political parties in the model formulated in Misra (2012). This was achieved by removing the constraint that the difference between the net rates of movement between the two political parties be sign definite. We defined two switching functions that depend on the size of each political party and some parameters. These functions generalize the Misra paper in which the net movement was assumed to be unidirectional, or in favour of a given political party. In addition to some results obtained in Misra (2012), additional information regarding how the behaviour of the population size is dependent on the switching parameters is demonstrated.

The inclusion of switching functions in this paper improved the Misra (2012) model. There are further aspects that can be considered in future. Among these we mention the possibility of including individual preferences in choosing a political party. Another aspect will be the improvement of the model by considering a non constant population. An interesting aspect to consider is the age structured model, in view of the fact that political parties often target the youths for the future sustainability of the parties. There is however a trade off between mathematical tractability and realism. Finally, one can also look at how media companies influence the dynamics of political parties.
